# Feasibility of preoperative planning using anatomical facsimile models for mandibular reconstruction

**DOI:** 10.1186/1746-160X-3-5

**Published:** 2007-01-15

**Authors:** Corrado Toro, Massimo Robiony, Fabio Costa, Nicoletta Zerman, Massimo Politi

**Affiliations:** 1 Department of Maxillofacial Surgery, University of Udine, Udine, Italy; 2 Institute of Oral Pathology, University of Ferrara, Ferrara, Italy

## Abstract

**Background:**

Functional and aesthetic mandibular reconstruction after ablative tumor surgery continues to be a challenge even after the introduction of microvascular bone transfer. Complex microvascular reconstruction of the resection site requires accurate preoperative planning. In the recent past, bone graft and fixation plates had to be reshaped during the operation by trial and error, often a time-consuming procedure. This paper outlines the possibilities and advantages of the clinical application of anatomical facsimile models in the preoperative planning of complex mandibular reconstructions after tumor resections.

**Methods:**

From 2003 to 2005, in the Department of Maxillofacial Surgery of the University of Udine, a protocol was applied with the preoperative realization of stereolithographic models for all the patients who underwent mandibular reconstruction with microvascular flaps. 24 stereolithographic models were realized prior to surgery before emimandibulectomy or segmental mandibulectomy. The titanium plates to be used for fixation were chosen and bent on the model preoperatively. The geometrical information of the virtual mandibular resections and of the stereolithographic models were used to choose the ideal flap and to contour the flap into an ideal neomandible when it was still pedicled before harvesting.

**Results:**

Good functional and aesthetic results were achieved. The surgical time was decreased on average by about 1.5 hours compared to the same surgical kind of procedures performed, in the same institution by the same surgical team, without the aforesaid protocol of planning.

**Conclusion:**

Producing virtual and stereolithographic models, and using them for preoperative planning substantially reduces operative time and difficulty of the operation during microvascular reconstruction of the mandible.

## Background

Medical rapid prototyping (MRP) is defined as the manufacture of dimensionally accurate physical models of human anatomy derived from medical image data using a variety of rapid prototyping (RP) technologies [1]. It has been applied to a range of medical specialties, including oral and maxillofacial surgery.

MRP was described originally by Mankowich et al in 1990 [2]. The development of the technique has been facilitated by improvements in medical imaging technology, computer hardware, three-dimensional image processing software, and the technology transfer of engineering methods into the field of surgery.

By using three-dimensional imaging a vast number of complex slice images can be quickly appreciated. The term 'three-dimensional', however, is not a truly accurate description of these images as they are still displayed on a radiological film or flat screen in only two dimensions. Computed Tomography offers volumetric information, which can be translated in three dimensional models. These models can be visualized but also exported to RP systems, that can produce these structures thanks to the rapidity and versatility of the technologies involved.

After resection of the mandible, reconstruction using a free vascularized bone graft has become the predominant treatment of choice [3].

Functional and aesthetic mandibular reconstruction after ablative tumor surgery continues to be a challenge even after the introduction of microvascular bone transfer.

One of the goals of mandibular reconstruction after tumor resection is a return to premorbid form and function. Complex microvascular reconstruction of the resection site requires accurate preoperative planning. Otherwise, postoperative surgical outcome often results in inadequate three dimensional mandibular shape and projection as well as disturbed function, thereby affecting the patient's quality of life [4].

Continuity defects created in the facial skeleton often result with treatment of certain pathologic conditions, most notably tumor ablation, osteoradionecrosis, and refractory osteomyelitis. The reconstruction of the mandible keeps being complicated for the maxillofacial surgeon, because functional and aesthetical properties must be accurately re-established. The bone graft must be the exact size and dimension of the defect, to assure a precise three-dimensional configuration of the mandible.

It is necessary to know the three-dimensional configuration of the mandibular defect, in order to choose the best donor site, and to prepare fixation rigid enough to withstand masticatory force.

Previously, the bone graft and the fixation plates had to be reshaped during the operation by trial and error, often a time-consuming procedure.

In these contexts, the availability of a copy of the real anatomy allows not only a planning but also, with the limitations due to the materials, a practical execution of the surgical operation. Nevertheless, the RP model presents also some disadvantages that can be reduced if the practical simulation is accompanied by a virtual simulation, performed on a digital model.

This paper outlines the possibilities and advantages of the clinical application of anatomical facsimile models in the preoperative planning of complex mandibular reconstructions after tumor resections.

## Methods

From 2003 to 2005, in the Department of Maxillofacial Surgery of the University of Udine, an operative protocol was applied with the preoperative realization of stereolithographic models for all the patients who underwent mandibular reconstruction with microvascular flap.

24 models were realized prior to surgery before emimandibulectomy or segmental mandibulectomy. The diagnoses were: squamous cell carcinoma (12 cases), ameloblastoma (9 cases), myxofibroma (1 case), aggressive juvenile ossifying fibroma (1 case), osteosarcoma (1 case).

The data acquisition has been performed using Computer Tomography (CT), the 3D model has been the result of Reverse Engineering (RE) practices based on image segmentation, and the real model has been produced using a RP technology called Stereolithography [5,6].

The digital data of the virtual reality were employed in the diagnostic phase and for the preliminary surgical planning. Moreover, virtual simulations on the 3D model have been obtained from image segmentation.

Data acquisition was performed by helical CT scanner single-slice *Toshiba Asteion*, or by four rowmultislice helical CT scanner *Toshiba Aquilion*.

The result of the CT, a sequence of gray images, constitutes the input, expressed in the form of raw data, for the RE activities, with the help of dedicated commercial software packages.

In the sequence of images representing the various sections, the anatomical structures can be identified on the basis of the gray level of the pixels. These anatomical regions are contoured using segmentation algorithms, and the three dimensional structure is reconstructed by generating skinning surfaces that join the resulting profiles. The surface representing the 3D model is described by way of a triangle mesh; this representation can be easily transferred to a Rapid Prototyping laboratory.

Stereolithography uses a liquid resin which is polymerized, layer by layer, by a UV laser beam which solidifies the region representing the area of the section. The part is built inside a vat full of liquid resin. When the building process is finished, the part is drained, the supports, which allowed the production of hanging parts, are easily removed and the polymerization is completed in a UV oven [7].

During the discussions with the surgeons the need for the simulation of the surgical procedure has been perceived not only on the model obtained from RP, but also on a digital model in a virtual context. In fact, from the first experiences with RP models, our surgical team appreciated the help obtainable from them, but underlined also their limitations, with particular accent on the fact that, once cut and manipulated, the stereolithography model is almost unusable. Therefore, it was necessary to find a method for simulating the surgical procedure on the virtual 3D model we had at our disposal. This model, representing the anatomical context relatively to the bone tissues, was available in the STL format generated by the image segmentation software. We used the software *Magic STL Fix *(V6.3.3.0 – Cimatron Ltd. Materialize N.V. – US) on an operating system *MS Windows 2000*.

The construction of the resin model begins by gathering data slices of 1 mm from CT scans. CT data are transferred to the Stereolithography machine (*SLA 3500 *– 3D Systems – Valencia, USA). Model fabrication starts with a tank full of liquid plastic and the data controlling computer. The platform is immersed in the liquid plastic and then raised to a level just below the surface of the viscous liquid photopolymer (Epoxy resin *Watershed 11120 *– DSM – Heerlen, NL). When a software-guided beam from a helium-cadmium laser strikes the surface of the liquid through a small series of adjustments, the plastic solidifies. After the first layer has been built, the platform lowers slightly in the tank and then is raised again. The guided laser once again strikes the liquid and polymerizes it. The dipping process is repeated to allow the layers to fuse. Each layer is polymerized at a thickness of approximately 0.125 mm [8]. Precise control of the movement of the platform, the viscosity of the liquid, and the position of the laser cause the solid plastic ridge to adhere to the platform. Once the model has been built, it is moved to an ultraviolet (UV) oven for postcuring. This design process can produce a 40-g resin replica in approximately 10 hours.

The titanium plates to be used for fixation were chosen and bent on the stereolithographic models preoperatively.

The data of the virtual resected mandibles were used to calculate the ideal position and angulation of osteotomies of the microvascular flaps in the three planes (x, y, z) to create an ideal "best-fit" of the neomandibles into the resection sites.

The geometrical information of the virtual mandibular resections and of the stereolithographic models was used to choose the ideal flap and to contour the flap into an ideal neomandible when it was still pedicled before harvesting. The preformed microvascular bone was then trasferred to the resection site without further osteotomy.

24 microvascular flaps were employed for the reconstructions: 19 iliac free flaps and 5 fibular free flaps.

All the operations were performed by the same double surgical team (contemporary mandibular resection and flap raising).

Orthopantomographies were performed immediately after surgery, at the end of the bone consolidation period (3 months after surgery), and at the time of the removal of the fixation plates (6 months after surgery).

## Case Presentations

### Case 1

**Figure 1:**
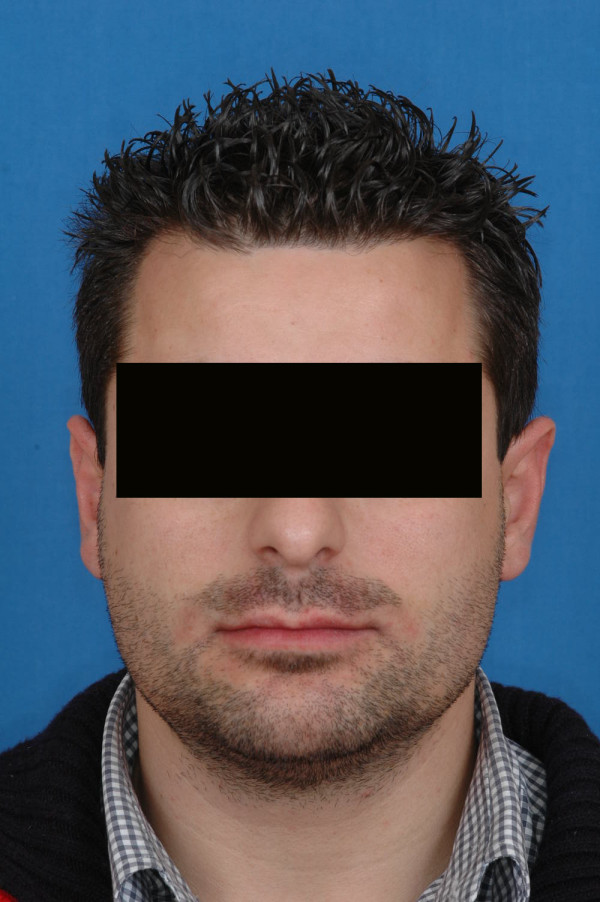
preoperative view of the patient n°1.

**Figure 2:**
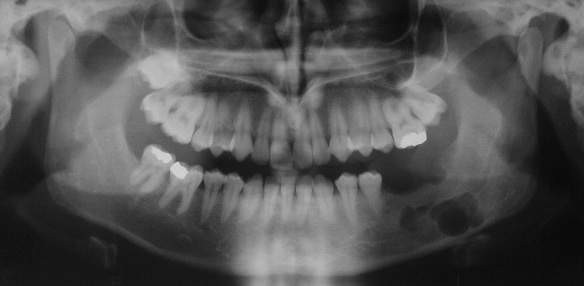
panoramic radiograph showing left mandibular lesion in patient n°1.

**Figure 3:**
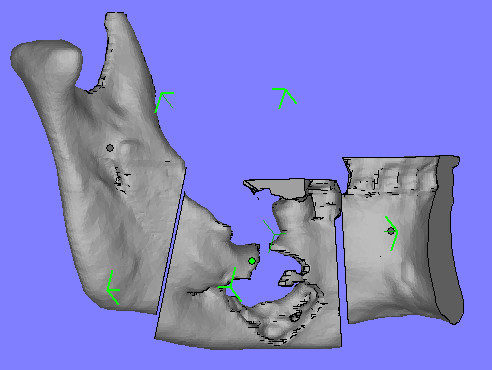
preoperative planning of the surgical resection on virtual reality with CAD software.

**Figure 4:**
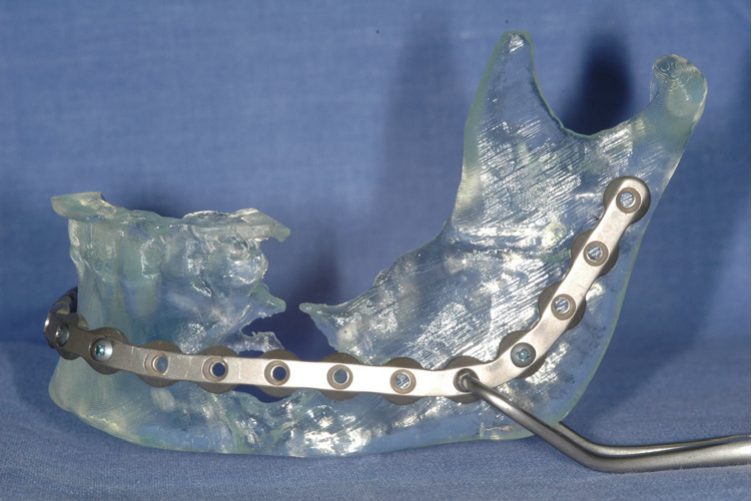
stereolithographic model and prebent reconstruction plate with adequate extension beyond area of projected resection.

A 28-year-old man was referred to our Clinic for treatment of a recurrence of ameloblastoma, previously treated in another institution (Fig. 1). Panoramic radiograph showed a multiloculated lesion on the left mandibular body (Fig. 2). The surgical procedure was first planned on the virtual digital model (Fig. 3), it helped us to measure exactly and to visualize the entity of the resection. A stereolithographic model of the left mandible was constructed. Prior to surgery, a mandibular reconstruction plate was pre-bent using the stereolithographic model as a reference. Screw placement was also planned and marked on the model, as well as screws length, which were recorded by measuring the thickness of the model at each plate hole (Fig. 4).

The focus of the treatment was to resect the tumor and preserve the form of the mandible. Before resection, the reconstruction plate was placed intraorally along the inferior border of the mandible, and screw holes were drilled to facilitate later fixation of the plate. The time spent in adapting the reconstruction plate was less than 5 minutes because no adjustments to the plate were necessary.

**Figure 5:**
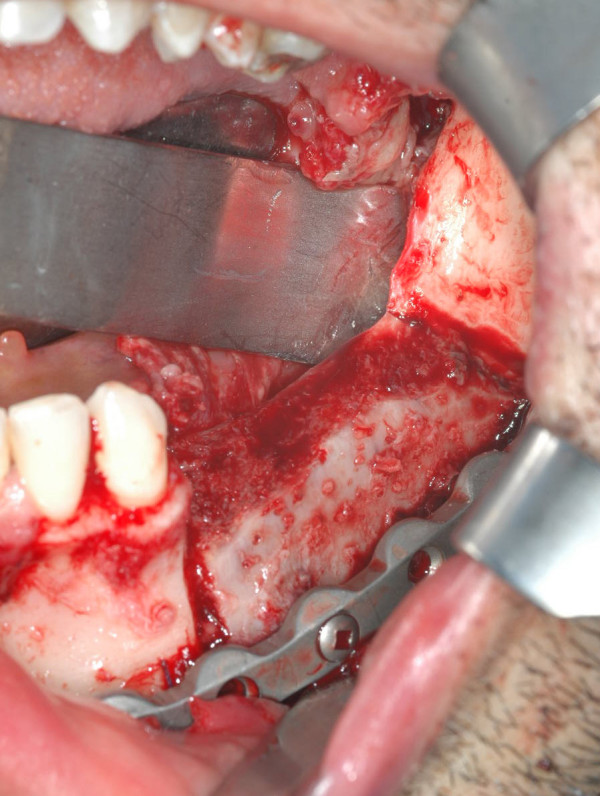
intraoperative view after the fixation of the microvascular iliac flap with the prebent plate.

**Figure 6:**
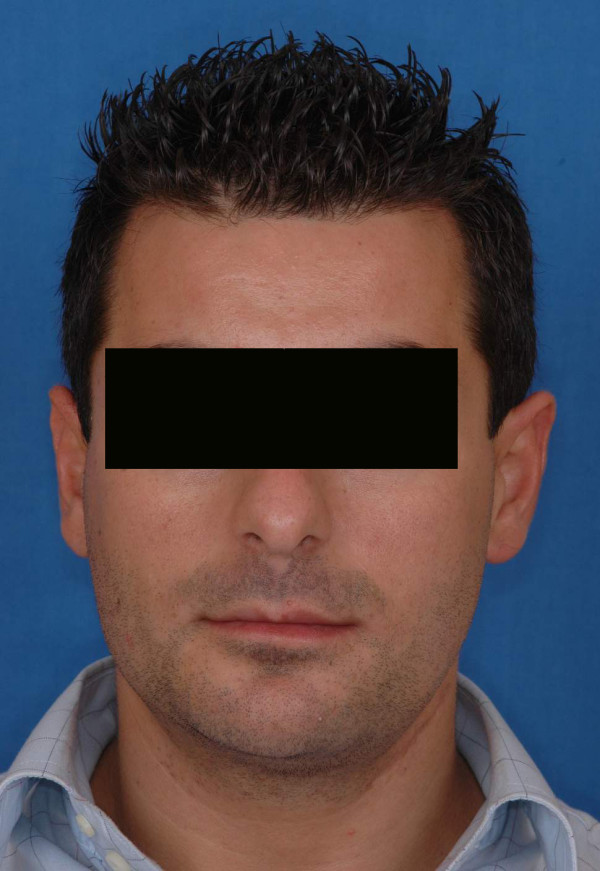
postoperative clinical view of the patient n°1, six months after surgery.

**Figure 7:**
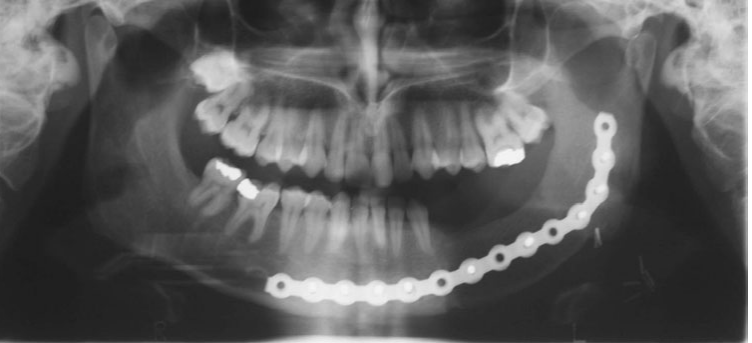
panoramic radiograph of patient n°1, six months after surgery.

**Figure 8:**
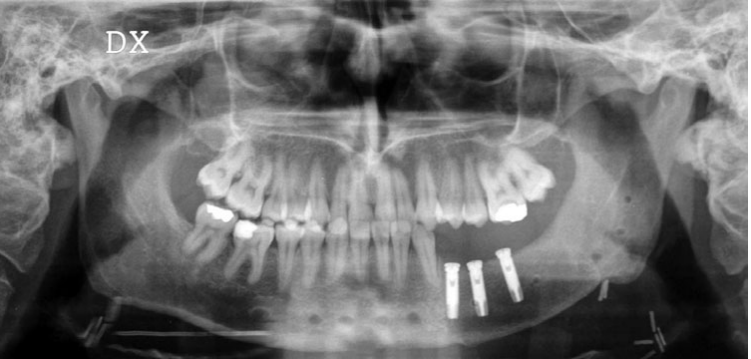
panoramic radiograph of patient n°1, six months after surgery, and after the plate removal and implants insertion.

The iliac microvascular flap was harvested and modelled using the dimensional data recorded during the preoperative planning on the digital model and on the stereolithographic model. The insertion of the flap in the osseus gap was easy and with excellent fit (Fig. 5). 6 months after the reconstruction, the plate was removed and implants were inserted (Fig. 6, 7, 8).

### Case 2

**Figure 9:**
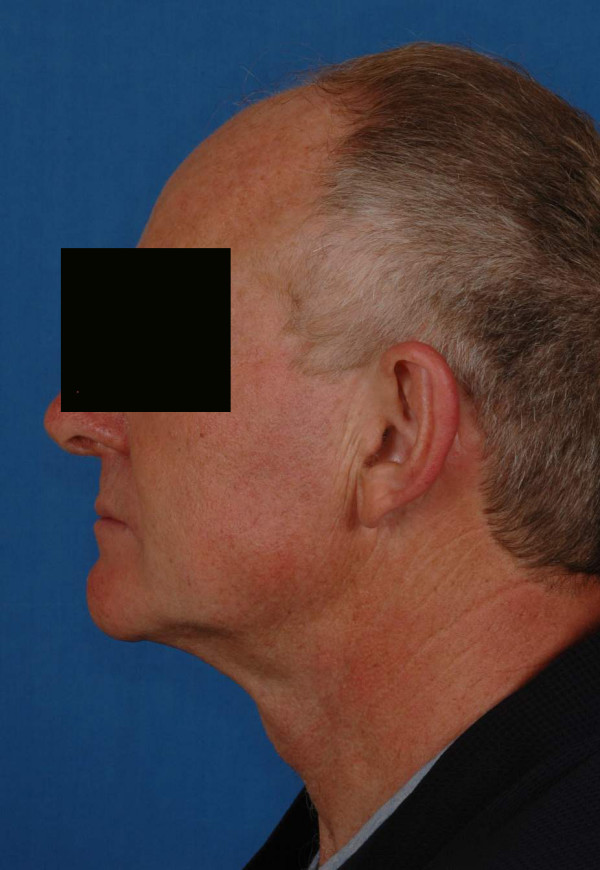
preoperative views of the patient n°2.

**Figure 10:**
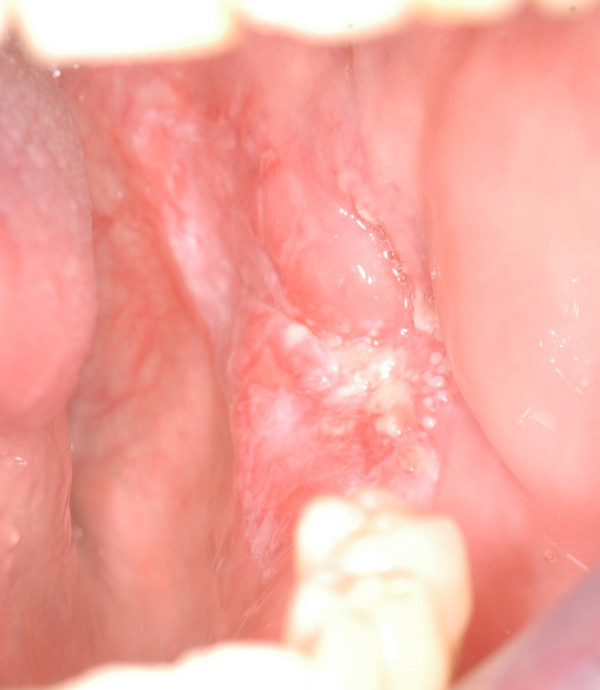
intraoral preoperative views of the patient n°2. A squamous-cell carcinoma of the left retromolar trigone invading the mandible.

**Figure 11:**
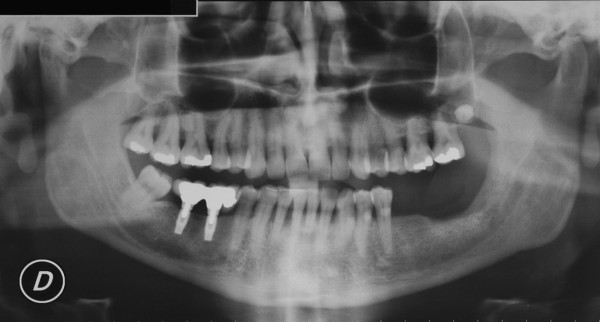
panoramic radiograph of patient n°2. Note the erosion of the bone on the left side of the mandible.

**Figure 12:**
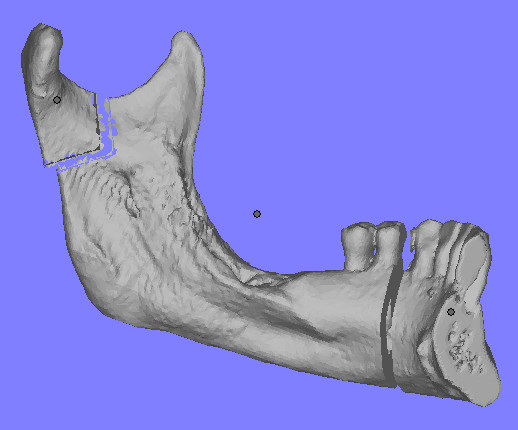
preoperative planning of the surgical resection on virtual reality. The macroscopic neoplastic involvement of the ramus imposed a wide resection.

**Figure 13:**
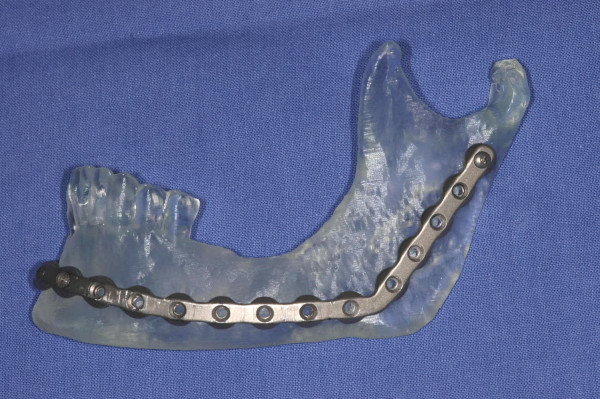
stereolithographic model of patient n°2. The model confirmed the macroscopic involvement of cortical bone. Holes were drilled and lenghts accurately measured and recorded.

The patient was a 50-year-old man who had a T4N0M0 squamous-cell carcinoma of the gingiva invading the left mandible (Fig 9, 10, 11). The planning was made according to the aforementioned protocol (Fig. 12, 13).

**Figure 14:**
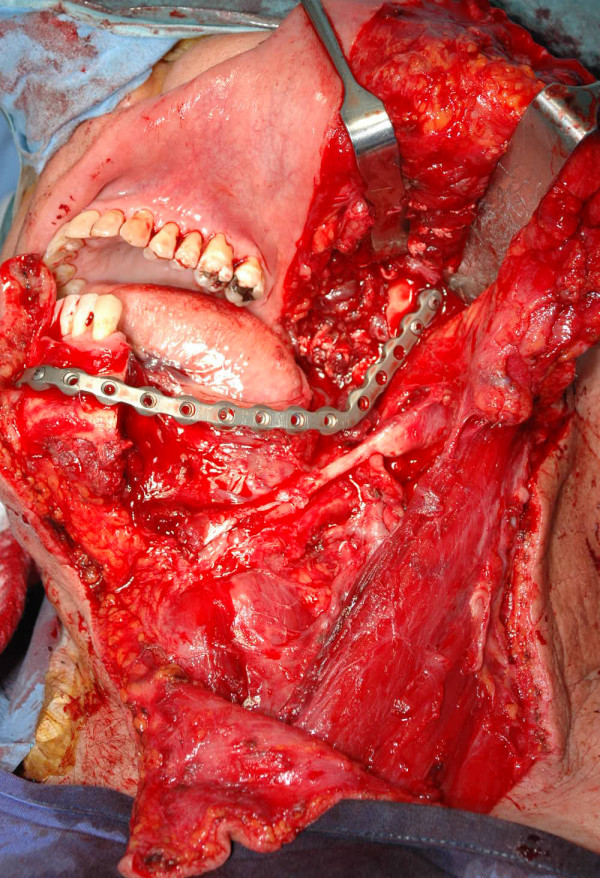
intraoperative view. An en-block resection with selective neck dissection was performed. Note the surgical defect and the excellent adaptation of the prebent plate, no modifications were necessary.

**Figure 15:**
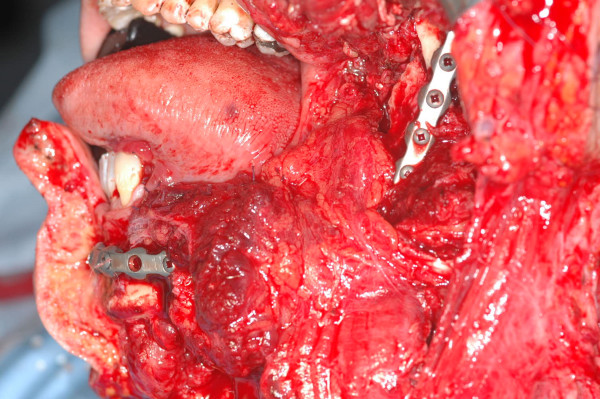
the microvascular osteo-muscolar iliac flap positioned. The internal-oblique muscle was rotated for the recostruction of the intraoral mucosa.

**Figure 16:**
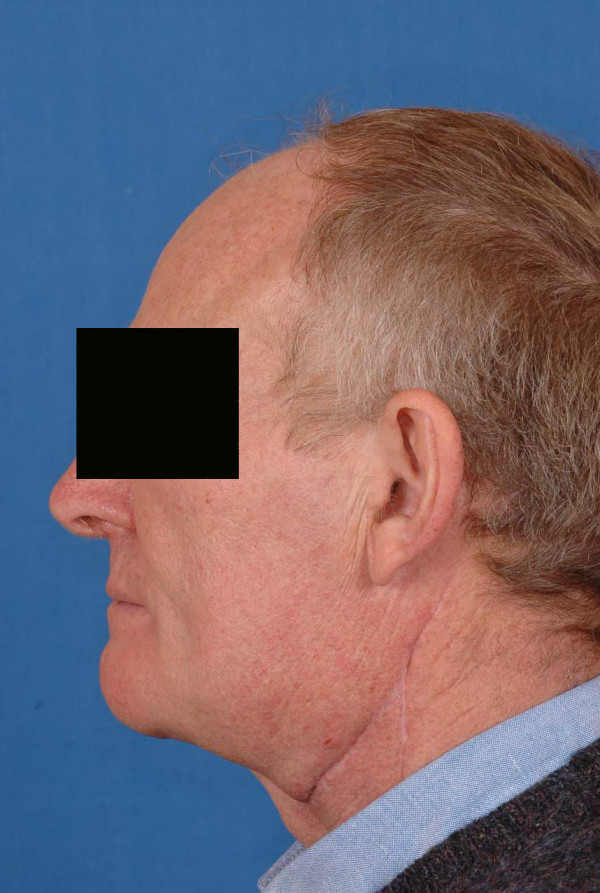
postoperative views of the patient n°2, 1 year after surgery.

**Figure 17:**
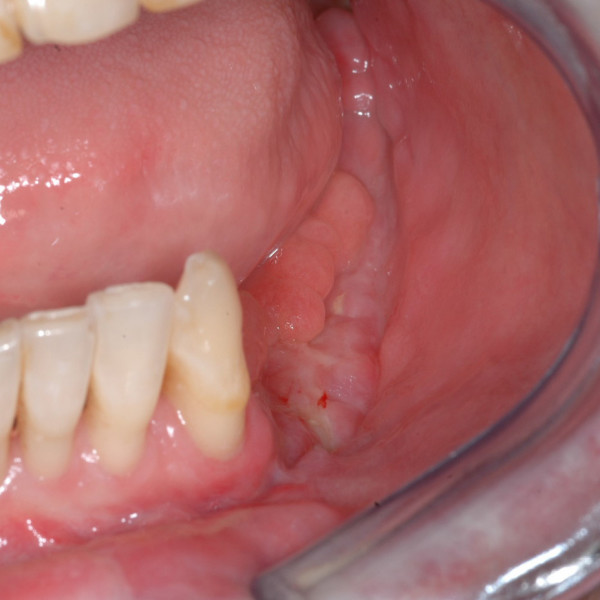
intraoral postoperative views of the patient n°2, 1 year after surgery.

**Figure 18:**
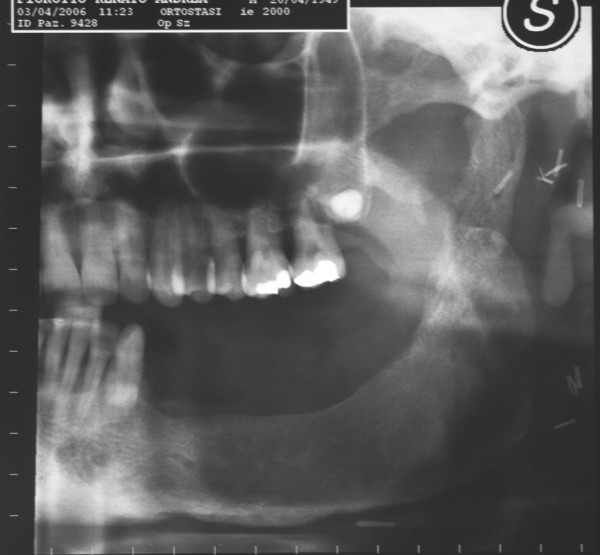
panoramic radiograph of patient n°2, 1 year after surgery.

Reconstruction was planned with a iliac microvascular osteo-muscolar flap with the use of a portion of the internal oblique muscle for the reconstruction of the intraoral mucosa. The resected specimen included the left mandible from premolars to the entire ramus, saving the condyle. The reconstruction plate was then placed; no intra-operative modifications were necessary (Fig. 14, 15). There were no signs of recurrence 1 year after surgery. The plate could be removed and the reconstruction appeared suitable for the use of bone-integrated dental implants (Fig. 16, 17, 18).

## Results

In all patients the intraoperative situation corresponded to the stereolithographic model.

The virtual and model planning acted as a guide to the length, the shape (in particular the mandibular angle), the height as well as the contour of the bone graft, thus minimizing the number of osteotomies.

There were 2 fibular flaps reexplorations, with 1 salvage and 1 failure (bone initially sectioned into 3 fragments), yielding an overall flap success rate of 95.8% (23 of 24).

Clinically and radiographically, there was nearly perfect symmetry of the reconstructed mandibles and undisturbed bone healing of the 23 survived flaps.

The mean anesthetic time (combined resection and reconstruction time) was 9 hours (range, 8 to 14). We reviewed our data on the precedents microvascular mandibular reconstructions performed since 2001 to 2003. Actual surgical time was decreased on average by about 1.5 hours compared to the same surgical kind of procedures performed, in the same Institution by the same surgical team, without the aforesaid protocol of planning.

Good functional and aesthetic results were achieved. Unforeseen complications such as malocclusion, evident asymmetry of the mandible, or poorly adapted plates were avoided.

No important functional sequelae were noted at the donor sites. One patient complained about limping for 2 months after surgery.

At the time of last follow-up, all 24 patients were alive. 22 patients have had endoosseus implants placed.

## Discussion

Construction of accurate anatomical replicas using modern digital design and manufacturing techniques can be an extremely useful method for capitalizing on imaging studies by transforming data into tangible objects. RP-generated anatomical models translate image data into solid replicas giving surgeons the means for tactile interaction with patient anatomy prior to an operation. Models can be highly effective for surgical planning, improving communication between medical experts and patients, and facilitating customization of treatment devices. In addition, treatment aids that incorporate patient-specific anatomical features and pre-planned treatment parameters such as drill trajectories or osteotomy planes give surgeons elegant and reliable tools to carry treatment designs from the computer to the operating room. Such tools and techniques can make it possible to manipulate existent patient anatomy to reflect idealized changes [9].

The clinical application of medical models was analyzed in a European multicenter study [10]. Results were collated from a questionnaire sent out to partners of the Phidias Network on each institution's use of stereolithography models. The 172 responses indicated the following range applications:

- To aid production of a surgical implant

- To improve surgical planning

- To act as an orienting aid during surgery

- To enhance diagnostic quality

- To be useful in preoperative simulation

- To achieve patient's agreement prior to surgery

- To prepare a template for resection.

3D models can be used to preshape metallic implants that will be used in surgery. The length of implant plates and the number and lengths of required screws can be planned before surgery [11].

It is also possible to plan osteotomies by performing a surgical rehearsal on the model itself. Such a rehearsal allows measurement of the displacement of all bone segments and anticipation of the size and the form of bone grafts; using these techniques requires the development of reference points so that sectioned portions of the model can be replaced into their original positions (eg, using plaster or dental stone to create a base around the uncut model) [12].

The models clearly show deformities or disease states and provide the surgeon with a mental image of the patient's anatomy that in turn decreases operative time and operative blood loss and improves the accuracy of plate and screw placement [13].

Manufacture of custom-made implants adjusted to a 3D model is foreseeable. Methyl methacrylate (acrylic) fabricated from models is already used as implant material during cranioplasty [14]. Nowadays microsurgical free tissue transfer allows reconstruction of the oromandibular area with improved functional and aesthetic results compared with other techniques. Vascularized bone flaps have become the preferred method for the reconstruction of composite mandibular defects [3,4].

Rose and colleagues first reported the use of high-tech 3D computer-generated models in facial reconstructions with vascularized grafts [15]. They described detailed preoperative plans, with less guesswork regarding size, contour, and orientation of the graft. Taylor also reported the usefulness of a 3D replica of a curved bone in mandibular reconstruction [16].

In head and neck reconstruction, in particular in mandibular defects, we need to know preoperatively the exact 3D structure of the bone and soft tissue. Contours of the face are composed of a balance of hard and soft tissues. The mandibular contour along the inferior border depends mainly on the shape of the mandible. Furthermore, in mandibular reconstruction, it is important to reconstruct the bone defect exactly to maintain normal occlusion.

In our experience, the use of an individual virtual and stereolithographic model proved to be a useful approach to plan the precise osteotomy sites and angulations, leading to a predictable shape of the reconstructed neomandible before the vascular pedicle was detached. This procedure significantly shortened the ischemic time of the graft as well as the duration of the operation because no additional contouring of the vascular bone graft is required before defect reconstruction.

While having a model of the deformed anatomy is instructive and can offer insight into surgical planning, the models must be manipulated (contoured using a surgical bur or a saw) to achieve symmetry if they are to be used to prebend reconstruction plates and guide bone grafting. For tumor surgery and reconstruction, the ability to manipulate the 3D images with computer software is very helpful in recreating symmetry of the jaw after resection [12].

In reconstruction with vascularized bone graft, there is the problem of how to fit the graft into the defect. The most appropriate site for the donor bone must be chosen carefully, in order to reshape the graft without injuring the vascular pedicle. It is time consuming to reshape and adjust the bone graft by trial and error during the operation. Simulation surgery, using a life-sized, solid model saves time and effort, thus contributing to a decrease in operating time [17,18].

Adapting a reconstruction plate intraoperatively, prior to tumor resection, would interfere with radical surgery [19]. Plates bent in simulation surgery are sterilized and can be used in the operative procedure. Fixation of the bone flap can be performed after microvascular anastomoses, because the exact shape and size have already been determined in the model surgery.

Although producing the models is expensive, using them for preoperative planning substantially reduces operative time and difficulty of the operation. Saving operative time is important because operating room costs average 30% to 40% of hospital expenses [18].

The costs for the stereolithograpic processing should rather contribute to the saving of money compared with costs of secondary corrections [20].

## Conclusion

In our series surgery proceeded according to the simulation with a relatively short operating time. We found that operating time decreased of about 1 – 1.5 hours in comparison to the same type of operation performed without the CT-guided stereolithography and virtual reality surgical planning.

An added benefit of a prebent reconstruction plate is that the resection can more easily be performed intraorally, thereby allowing a more conservative surgical approach.

The greatest disadvantages of using SLA models are the time and cost involved in making the models. In our region the cost of a mandibular model fabrication can range from € 200 to € 400. Improvements in the RE – RP apparatus and in the materials used to fabricate the models, and increase in its clinical applications should decrease the cost of the models.

## Competing interests

All the authors state that there any non-financial competing interests (political, personal, religious, ideological, academic, intellectual, commercial or any other) to declare in relation to this manuscript.

## Authors' contributions

CT performed the microsurgical reconstructions; he have made substantial contributions to conception and design of the paper.

MR performed the surgical removal of the reported tumors; he revised the manuscript critically for important intellectual content.

FC have made acquisition, analysis and interpretation of data.

NZ have been involved in drafting the manuscript and in revising it.

MP have given final approval of the version to be published.

All authors read and approved the final manuscript.
